# Supplementary material for: Analysis on the structure effect of marine fishery total factor productivity under high-quality development in China
Source: PLoS One. 2021 Nov 24;16(11):e0259853. doi: 10.1371/journal.pone.0259853 (PMC8612771; doi:10.1371/journal.pone.0259853)
Supplement: Attachment — Submitted filename: Response to Reviewers.doc [file pone.0259853.s002.doc]

Dear editors

Thank you very much for the editors and review experts for their suggestions for this paper. First, I reply to the amendment suggestions one by one. The content is as follows:

1.Please ensure that your manuscript meets PLOS ONE's style requirements, including those for file naming.

**Reply: The paper has been revised according to the format requirements.**

2. Thank you for stating the following financial disclosure:
   “Project Support: Youth Project of Natural Science Foundation of Guangxi Science and Technology Department (Number: 2018GXNSFBA050010); Youth Project of National Social Science Foundation of China ( Number: 19CJY023); Guangxi Key R & D Plan ( Number: Guike AB1850023)”

**Reply:**

Youth Project of Natural Science Foundation of Guangxi Science and Technology Department (Number: 2018GXNSFBA050010) and Youth Project of National Social Science Foundation of China ( Number: 19CJY023) provide support in study design and analysis.The paper is one of the phased research results of this project(Youth Project of Natural Science Foundation of Guangxi Science and Technology Department (Number: 2018GXNSFBA050010).

Guangxi Key R & D Plan ( Number: Guike AB1850023)provided basic materials for writing the thesis (such as marine fishery development data).

3. In your Data Availability statement, you have not specified where the minimal data set underlying the results described in your manuscript can be found. PLOS defines a study's minimal data set as the underlying data used to reach the conclusions drawn in the manuscript and any additional data required to replicate the reported study findings in their entirety. All PLOS journals require that the minimal data set be made fully available.

**Reply: For the data involved in the paper, the author has sent it to the journal in the form of a file (excel)**

4.Please ensure that you refer the all Figure (1-7) in your text as, if accepted, production will need this reference to link the reader to the figure.

**Reply: It has been verified, and the name of the relevant figure is marked in the text.**

5.In order to improve the quality of your manuscript, we especially suggest that you include citations in the discussion and land a little on the conclusions according to the objective pursued in the research.

**Reply: Adding this reference:**

*Dios-Palomares, R. (2015). Analysis of the Efficiency of Farming Systems in Latin America and the Caribbean Considering Environmental Issues. Revista Cientifica-Facultad de Ciencias Veterinarias, 25(1). Available in publons.com/p/3106827/*

Reviewer #2:

1. the abstract is not well written, and it needs an conclusion and sussestion;

**Reply:The abstract has been revised and refined. We newly added essay viewpoint. as follows:**

“*We believe that simply pursuing the advanced evolution of the industrial structure is not conducive to sustainable development of fisherie. Under the pursuit of the rationalization of the marine fishery industry structure, by promoting the coordinated evolution of marine fisheries advancement, aquaculture-catching structure and other factors, the "structural dividend" effect can be enhanced and the fishery can achieve sustainable development.*”

2. in the Question raising and literature review (part 1), there are no references for the datas;

**Reply: Added data sources in the form of footnotes,as follows:**

*Data source: "China Fishery Statistical Yearbook". If there is no special description in the following text, the data source is the same.*

3. there are two many simple figures in the manuscript, and many freshwater data is needless, in the figure 4, freshwater aquaculture has been written to mreshwater aquaculture;

**Reply: The freshwater data has been deleted, and this figure has been updated. as follows.**

4. I can not found any references in the discussion and Conclusion and suggestions, please added it.

**Reply: Based on the analysis and conclusions of the research results of the thesis, relevant references have been added**

*Han YH, Zhang F, Huang LX, Peng KM, Wang XB. Does industrial upgrading promote eco-efficiency? ─A panel space estimation based on Chinese evidence. Energy Policy. 2021; 154: 112286.doi: org/10.1016/j.enpol.2021.112286.*

*Zhou Y, Kong Y, Sha J, Wang HK. The role of industrial structure upgrades in eco-efficiency evolution: Spatial correlation and spillover effects. Sci Total Environ. 2019; 687: 1327-1336.doi: 10.1016/j.scitotenv.2019.06.182.*

*Xiang XM. The transformation and upgrading path of my country's marine fishery from the perspective of agricultural supply-side structural reform. Social Sciences in Guangdong. 2017; (05):23-29.*

*Gan C, Zheng RG, Yu DF. An empirical study on the effects of industrial structure on economic growth and fluctuations in China. Economic Research Journal. 2011; 46(05):4-16,31. doi: 10.1093/hmg/ddr440.*

*Zhang Y, J Ji. The decoupling and influencing factors analysis of blue granary Eco-Economy system. Journal of Agrotechnical Economics. 2020; (04):94-106. doi: 10.13246/j.cnki.jae.2020.04.006.*

*Yang L, Su X. Research on the objectives and implementation paths of the optimization and upgrading of the marine fishery industrial structure from the perspective of industrial ecology. Issues in Agricultural Economy. 2010; 31(10): 99-105.doi: 10.13246/j.cnki.iae.2010.10.011.*

*Shao QL, Chen LJ, Zhong RY, Weng HT. Marine economic growth, technological innovation, and industrial upgrading: A vector error correction model for China. Ocean & Coastal Management. 2021; 200: 105481.doi: 10.1016/j.ocecoaman.2020.105481.*

*Lin XH, Zheng L, Li WW. Measurement of the contributions of science and technology to the marine fisheries industry in the coastal regions of China. Marine Policy. 2019; 108: 103647.doi: 10.1016/j.marpol.2019.103647.*
